# Hydropersulfides (RSSH) and Nitric Oxide (NO) Signaling: Possible Effects on S-Nitrosothiols (RS-NO)

**DOI:** 10.3390/antiox11010169

**Published:** 2022-01-16

**Authors:** Jon M. Fukuto, Cristina Perez-Ternero, Jessica Zarenkiewicz, Joseph Lin, Adrian J. Hobbs, John P. Toscano

**Affiliations:** 1 Department of Chemistry, Johns Hopkins University, Baltimore, MD 21218, USA; jzarenk1@jhu.edu; 2 Department of Chemistry, Sonoma State University, Rohnert Park, CA 94928, USA; 3 William Harvey Research Institute, Barts & The London School of Medicine, Queen Mary University of London, Charterhouse Square, London EC1M 6BQ, UK; c.perez-ternero@qmul.ac.uk; 4 Department of Biology, Sonoma State University, Rohnert Park, CA 94928, USA

**Keywords:** S-nitrosothiol, hydropersulfide, nitric oxide, nitroxyl, perthiyl radical

## Abstract

S-Nitrosothiol (RS-NO) formation in proteins and peptides have been implicated as factors in the etiology of many diseases and as possible regulators of thiol protein function. They have also been proposed as possible storage forms of nitric oxide (NO). However, despite their proposed functions/roles, there appears to be little consensus regarding the physiological mechanisms of RS-NO formation and degradation. Hydropersulfides (RSSH) have recently been discovered as endogenously generated species with unique reactivity. One important reaction of RSSH is with RS-NO, which leads to the degradation of RS-NO as well as the release of NO. Thus, it can be speculated that RSSH can be a factor in the regulation of steady-state RS-NO levels, and therefore may be important in RS-NO (patho)physiology. Moreover, RSSH-mediated NO release from RS-NO may be a possible mechanism allowing RS-NO to serve as a storage form of NO.

## 1. Introduction

Nitric oxide (NO) is an endogenously generated regulator of vascular tone via the activation of the enzyme soluble guanylyl cyclase (sGC), which converts guanosine-5′- triphosphate (GTP) to cyclic guanosine-3′,5′-monophosphate (cGMP), leading to smooth muscle relaxation [1,2]. This NO/sGC/cGMP signaling pathway represents the primary mechanism of NO-mediated physiology. The discovery of a signaling pathway driven by the regulated enzymatic synthesis of a small, readily diffusible molecule, NO, led to interest in other small, endogenously synthesized di- or tri-atomic molecules as potential physiological signaling agents (e.g., carbon monoxide (CO) and hydrogen sulfide (H_2_S)). It is now established that the small-molecule species NO, CO, and H_2_S have important physiological functions, and much of this signaling occurs in an integrated fashion [3,4]. Indeed, these three molecules, along with dioxygen (O_2_), now constitute a special class of endogenous, small (di- or tri-atomic), signaling species (sometimes referred to as “gasotransmitters”—an inadvisable and misleading term since they are all aqueous solutes, and not gases, when they act physiologically). Among the myriad physiological functions of NO, CO, and H_2_S (along with derived species) are the regulation of vascular tone, immune response, central nervous system function, wound healing, and the regulation of respiration, just to name a few [5,6]. However, unlike NO and the well-characterized NO/sGC/cGMP pathway, the exact mechanism(s) associated with CO or H_2_S signaling activity have not been nearly as established or defined.

## 2. Physiological Relationship between H_2_S and NO

Numerous reports indicate a special connection between the signaling actions of NO and H_2_S, especially in the cardiovascular system [7,8]. For example, low concentrations of H_2_S significantly enhance smooth muscle relaxation induced by NO [9]. Consistent with this finding, H_2_S is capable of increasing NO production in endothelial cells, possibly via stimulation of mechanisms responsible for the phosphorylation (and activation) of endothelial nitric oxide synthase (eNOS, NOS3) [10]. Further reports of the effects of H_2_S on NO signaling include H_2_S-mediated inhibition of the phosphodiesterase PDE5, which would otherwise degrade the NO-derived second messenger cGMP [11], inhibition of proline-rich tyrosine kinase 2 by H_2_S, which would otherwise phosphorylate and inhibit eNOS [12], and stimulation of protein kinase G (PKG), a target for cGMP-mediated activity [13]. Although it appears that H_2_S has a clear synergistic relationship with NO, which may involve numerous targets in the NO/sGC/cGMP pathway, the chemical mechanisms responsible for these effects are not well understood and it is likely that other interactions exist by which H_2_S (or especially derived species) can affect NO-signaling processes.

## 3. S-Nitrosothiols (RS-NOs) and NO Signaling

Although the primary physiological target of NO-mediated signaling is sGC (*vide supra*), NO has also been proposed to affect the function of proteins via the modification of protein cysteine residues forming a RS-NO function [among many reviews are [14,15,16]]. The generation of a RS-NO functional group on a protein or peptide starting from NO and a thiol (RSH) under physiological conditions is not chemically straightforward and the mechanism(s) by which this occurs remain unclear; physiological mechanisms of RS-NO degradation are equally ill-defined. That is, it is clear that RS-NO species are prevalent in biology, but the mechanisms of their formation and degradation as well as the regulation of these processes remain elusive. Despite the lack of detailed understanding of the specific physiological mechanisms responsible for their formation and degradation, RS-NO generation has been touted as a cell signaling process analogous to phosphorylation [14,17]. Anointing RS-NO formation (sometimes questionably referred to as S-nitrosylation) in proteins as being analogous to phosphorylation as a physiological signaling paradigm seems premature at this time, especially since the specificity and regulation of phosphorylation via kinases and phosphatases are so well-defined and established [18] whereas an understanding of RS-NO formation/degradation is not nearly as complete [19]. To be sure, chemical mechanisms for the generation and degradation of RS-NO species have been proposed (*vide infra*), but clearly much more work will need to be done before any of these processes will be generally accepted to the degree that kinases and phosphatases are known to specifically control and regulate protein phosphorylation.

Regardless of whether RS-NO formation is analogous to phosphorylation as a signaling phenomenon, numerous reports indicate that the generation of RS-NOs on specific proteins can have physiological effects, both positive and negative (and/or opposing). Using reports of effects on inflammation as examples, it is apparent that RS-NO formation can potentially alter inflammatory signaling in opposite directions. For example, RS-NO formation on cysteines in the hydrophobic region of the surfactant protein-D (SP-D) results in the activation of inflammatory signaling via NF-κβ activation [20]. On the other hand, RS-NO formation on inhibitor of nuclear factor κβ (IKκβ) leads to inhibition of NF-κβ activation [21]. Furthermore, RS-NO formation of the signal transducer and activator of transcription 3 (STAT3) results in a decrease in inflammatory signaling [22], whereas RS-NO formation on the NAD^+^-dependent deacetylase Sirt1 results in inhibition and ultimately in the activation of NF-κβ/inflammatory signaling [23]. These few examples (among many others, e.g., RS-NO proteins and neurodegeneration [24]) indicate that RS-NO formation can have at times opposing effects on the same general pathways, depending on the proteins affected. Thus, elucidation of the mechanisms of formation/degradation that account for protein specificity become of paramount importance in understanding the possible regulation of signaling pathways.

## 4. Possible Mechanisms of Formation of RS-NO

As is evident from Reaction (1), the formation of a RS-NO species from RSH and NO requires a one-electron oxidation.

RSH + NO → RS-NO + e^−^ + H^+^, (1)

**Figure 1:**
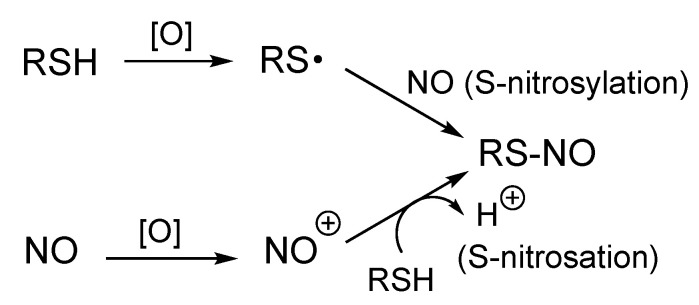
Possible pathways for the formation of RS-NO from RSH and NO. [O] denotes a one-electron oxidation.

The requisite one-electron oxidation step can occur on either reactant, NO or RSH, prior to (or during) RS-NO formation. One-electron oxidation of RS^−^/RSH gives the corresponding thiyl radical (RS^•^), which can directly couple with NO (a paramagnetic species with one unpaired electron) to give RS-NO (this coupling reaction can be termed ‘S-nitrosylation’ since it directly utilizes NO [19]). Alternatively, one-electron oxidation of NO generates a nitrosonium ion (NO^+^), which can nitrosate RSH, giving RS-NO (this reaction is considered as an ‘S-nitrosation’ since it involves NO^+^ [19]) (Figure 1).

Regardless of how RS-NO is made, to truly understand the physiological mechanism, it is essential that the one electron that must be removed is accounted for (i.e., that the electron acceptor is characterized and reasonable). To date, there have been several mechanisms proposed that can result in RS-NO generation (from NO and RSH) under physiological conditions. One highly discussed and considered mechanism requires an initial reaction of NO with O_2_, leading to the eventual generation of a nitrosating agent (e.g., N_2_O_3_ or NO_2_) [25,26,27] (Reactions (2)–(4)).

2NO + O_2_ → (N_2_O_4_) → 2NO_2_, (2)

NO_2_ + NO → N_2_O_3_, (3)

N_2_O_3_ + RSH → RS-NO + NO_2_^−^ + H^+^, (4)

Although this series of reactions can lead to the generation of RS-NO from NO and RSH, the kinetics makes this process difficult and slow (at least at typical physiological levels of NO, ≤1 micromolar). The overall kinetics of RS-NO formation is dependent on the formation of N_2_O_4_, the precursor to NO_2_ (Reaction (2)) and N_2_O_3_ (Reaction (3)). Significantly, both NO_2_ and N_2_O_3_ can generate a RS-NO species, but these are very slow processes at physiological NO levels due to the second order dependence on NO concentration (t_1/2_ > 7 min) [27]. One possible caveat, however, is that NO and O_2_ are at higher levels in hydrophobic membranes, making this chemistry somewhat more viable in these environments [28].

Another mechanism that can lead to a RS-NO species from NO and RSH is via a metal-mediated process. For example, reductive nitrosation can occur at ferric (Fe^III^) centers (e.g., ferric-heme proteins with an open coordination site) capable of binding NO (Reactions (5) and (6)).

Fe^III^ + NO → [Fe^III^-NO ←→ Fe^II^-NO^+^], (5)

[Fe^III^-NO ←→ Fe^II^-NO^+^] + RSH → RS-NO + Fe^II^ + H^+^, (6)

Fe^II^ + NO → Fe^II^-NO, (7)

In this process, the ferric ion coordinates and accepts (at least partially) an electron from NO, generating a species with a “NO^+^-like” character, which is capable of nitrosating RSH as well as other nucleophilic species [29,30,31]. In these systems, the ferrous iron (Fe^II^) species generated in Reaction (6) is often capable of binding to NO, forming a stable ferrous nitrosyl adduct (Fe^II^-NO, Reaction (7)). It is shown in Figure 1 that thiols can be nitrosated by “NO^+^”. It needs to be emphasized that free NO^+^ will not exist (has no appreciable lifetime) under biological conditions as it is much too electrophilic and will immediately react with water. However, Fe^II^-NO^+^ species (Reaction (5)) have mild NO^+^-like character and can nitrosate thiols under biological conditions. Regardless, this chemistry is highly dependent on having a redox active, NO-binding metal in close proximity to a reactive RSH in order to form RS-NO. This is especially true since the Fe^II^-NO^+^ species can react with many other nucleophiles (including water) and is not specific for RSH nitrosation. Thus, the specificity of RS-NO formation will be highly dependent on the location of the appropriate metal (e.g., Fe^III^), the proximity of NO biosynthesis, and its juxtaposition to a reactive RSH.

Another possible mechanism for RS-NO formation is via the oxidation of RSH to the corresponding thiyl radical species (RS^•^), which can directly and rapidly couple with NO (Figure 1). RS^•^ species are generally potent oxidants (ε’ = 0.92 V for the RS^•^/RS^−^ couple [32]) and therefore are not readily/easily formed in biological environments. Although generation of RS^•^ in some enzymes (e.g., ribonucleotide reductase) is well-established [33] and required to perform essential oxidation chemistry (e.g., DNA biosynthesis), general and non-specific formation of RS^•^ can have toxicological consequences due to its inherent and indiscriminate oxidizing nature [34,35]. Thus, it seems unlikely that this chemistry represents a general mechanism for biological RS-NO formation (although in specific cases it may well be viable).

Clearly there are several possible mechanisms of RS-NO generation in biological systems (discussed above) and currently there appears to be no consensus regarding the relevance or prevalence of these pathways. Lancaster [36] speculates that all of the pathways discussed can be relevant, depending on the biological environment and nature of the RSH species. Importantly, however, these processes are oxidative in nature and more likely to occur under oxidizing conditions (or under cellular oxidative stress). This aspect of RS-NO generation will become important later in this discussion.

## 5. RS-NO Degradation Pathways

Similar to the level of understanding of RS-NO biosynthesis, there are known mechanisms of RS-NO degradation, but the physiological relevance of each of these pathways remains to be established. One pathway involves reduced metals, especially Cu^I^ [37] (Reaction (8)).

RS-NO + Cu^I^ → RS^−^ + NO + Cu^II^, (8)

This reaction can be catalytic in copper if the Cu^II^ formed in Reaction (8) is reduced back to Cu^I^. Interestingly, this chemistry appears to be optimum for copper as other biologically relevant metal ions such as Mg^2+^, Ni^2+^, Co^2+^, Mn^2+^, Cr^3+^, or Fe^3+^ exhibit no catalytic activity (although Fe^2+^ can have a small level of catalytic activity) [38]. Although this process will lead to the liberation of NO and RSH from RS-NO, to date there is no evidence of any metal (e.g., copper) containing redox-metalloprotein capable of readily performing this chemistry with a variety of RS-NO species (e.g., with varied RS-NO proteins). Moreover, freely available intracellular Cu^I^ (not sequestered or bound to a protein) is virtually non-existent [39] and therefore relying on Reaction (8) to occur based on levels of free Cu^I^ is extremely problematic. Thus, Reaction (8) may be responsible for artifactual trace metal-mediated decomposition of RS-NO species, but may not be as relevant to the general physiological degradation of RS-NO species.

Another possible mechanism of RS-NO degradation is via photolysis of the S–N bond (Reaction (9)) [37,40].

RS-NO + hν → RS^•^ + NO, (9)

2RS^•^ → RSSR, (10)

Under purely chemical conditions (lacking other possible reactants), the RS· formed in Reaction (9) will readily dimerize to the disulfide, RSSR (Reaction (10)). Needless to say, this chemistry is of limited biological relevance and will be relevant only to tissues exposed to light. For example, skin exposed to light (420–453 nm) results in increased cutaneous blood flow, possibly the result of photochemical NO generation from endogenous RS-NO species [41]. As mentioned previously, the RS· formed in this reaction is highly oxidizing and presents a possible indiscriminate oxidant capable of tissue damage. Thus, this chemistry may have pharmacological and therapeutic applications whereby NO release from RS-NO will only occur where light is administered, but its general physiological relevance seems unlikely.

Importantly, several dehydrogenase enzymes have been found to be capable of reducing the RS-NO of glutathione (GSNO). Jensen and co-workers reported that rat alcohol dehydrogenase class III (also referred to as glutathione-dependent formaldehyde dehydrogenase) can reduce GSNO to several products including glutathione sulfinamide (GS(O)NH_2_, which can hydrolyze to sulfinic acid (GS(O)OH) and ammonia (NH_3_) and glutathione disulfide (GSSG)/hydroxylamine (NH_2_OH), depending on the conditions [42]. In either case, the “GS” component of GS-NO is converted to a non-reducing, less reactive form as the thiol functional group is lost. Liu and co-workers also reported that enzymes isolated from a variety of sources (and also identified as alcohol dehydrogenase class III enzymes) are capable of converting GS-NO to GSH and ammonia (NH_3_) as primary products [43]. This group also reported that these enzymes are responsible for the majority of GS-NO degradation in mouse liver. These processes appear to be fairly specific for GS-NO as other RS-NOs were found to be poor substrates. These enzymes, which were previously characterized by their dehydrogenase activities with other substrates and named accordingly, have also been referred to as S-nitrosoglutathione reductases (GSNOR). Since GS-NO may be involved in an equilibrium with many protein RS-NOs via a transnitrosation reaction (discussed immediately below), the lowering of GSNO via these processes can lower overall RS-NO levels in cells [43]. However, if there are protein RS-NOs that are not in ready equilibrium with GS-NO, they may be resistant to the RS-NO lowering effect caused by GS-NO degradation.

Another mechanism of RS-NO degradation (alluded to directly above) that does not directly liberate NO is transnitrosation (Reaction (11)) [44,45].

RS-NO + R’SH → RSH + R’S-NO,(11)

In this reaction, the nitrosonium group is simply transferred from one thiol to another. It is important to reiterate that unlike the previously discussed mechanisms of RS-NO degradation, this reaction does not destroy the RS-NO functional group as it simply transfers NO^+^ to another R’SH, resulting in another RS’-NO species. Both the kinetics and thermodynamics of this process have been examined and it appears that this reaction is not generally rapid (*k* < 150 M^−1^s^−1^) with fairly widely ranging equilibrium values [46]. Since the transnitrosation reaction relies on the nucleophilic attack of a thiolate anion (RS^−^) on the electrophilic nitrogen atom of RS-NO, the p*K*_a_ of the attacking RSH species is an important factor in determining which RSH (or RS^−^) participates in this chemistry.

The reaction between RSH and RSNO has another fate besides the transfer of NO^+^ (as shown in Reaction (11)). Others have reported that along with possible attack of a nucleophilic RS^−^ on the nitrogen atom of RS-NO, nucleophilic attack at the sulfur atom is also possible, generating the corresponding disulfide and nitroxyl (HNO), (Reaction (12)) [47,48]. This pathway can be referred to as “S-thiolation” due to the formation of the disulfide product (a term that can also be used for other reactions whereby an oxidized thiol species is converted to a disulfide via reaction with a thiol).

RS-NO + R’SH → R’SSR + HNO,(12)

The disulfide formed in this chemistry can then be readily reduced back to the corresponding RSH species in biological systems. Unlike several of the previously mentioned RS-NO degradation pathways that either produce NO (Cu^I^-mediated reduction or photolytic decomposition) or transfer NO^+^ to another R’SH regenerating another R’S-NO, S-thiolation, Reaction (12) produces another nitrogen oxide species, HNO. Importantly, the chemical biology of HNO is distinct from NO, although HNO and NO can interact at similar biological targets [49]. One of the most important targets for the actions of HNO are thiols/thiol proteins. HNO is particularly thiophilic (reacts readily with RSH), and has two primary reaction pathways depending on the reaction conditions. HNO can react with an excess of RSH to generate a disulfide and hydroxylamine (Reaction (13)) or can react with low concentrations of RSH to give a sulfinamide, which can hydrolyze to the corresponding sulfinic acid (Reaction (14)) (for a review of the chemistry of HNO, see [50]).

2RSH + HNO → RSSR + NH_2_OH, (13)

RSH + HNO → RS(O)NH_2_ → RS(O)O^−^ + NH_3_, (14)

Importantly, Reaction (13) is considered to be a biologically readily reversible RSH modification since RSSR can be easily reduced back to the RSH species, while Reaction (14) generates a thiol modification (either the sulfinamide or sulfinic acid), which for the majority of proteins represents an essentially irreversible oxidative modification (or at least reversal is very slow). Thus, degradation pathways of RS-NO species that lead to HNO formation can potentially result in further RSH modifications with varying fates. It is also noteworthy that HNO is capable of being converted to NO via a simple one-electron oxidation (Reaction (15)), providing another, albeit circuitous, pathway for the generation of NO from RS-NO degradation.

HNO → NO + e^−^ + H^+^, (15)

Further commentary on HNO chemistry/physiology was beyond the scope of this discussion, however, suffice to say that HNO generation from RS-NO degradation is potentially complex with multiple pathways available and multiple fates possible.

**Figure 2:**
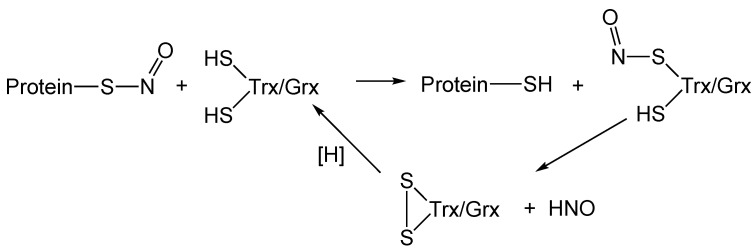
Possible mechanism for the Trx/Grx-mediated denitrozation of a protein RS-NO. [H] represents known biological pathways for RSSR reduction to the corresponding RSH.

There are numerous enzyme systems designed to reduce oxidized thiol proteins (e.g., protein disulfides) to maintain proper cellular redox homeostasis (in this context, the proper ratio of oxidized/reduced thiol proteins). Since RS-NO is an oxidized thiol species, it is not surprising that at least some of these enzyme systems are capable of reducing RS-NO to RSH. Among these enzyme systems, thioredoxin (Trx) and glutaredoxin (Grx) have been reported to be capable of performing this biochemistry [51,52]. Both Trx and Grx have been proposed to accept the NO^+^ function from an S-nitrosated protein via transnitrosation (Reaction (11)) followed by eventual conversion of the S-nitroso Trx or S-nitroso Grx to a disulfide and release of HNO (Reaction (12)). Reduction of the protein disulfides to the active thiols then completes the catalytic cycle [53] (Figure 2).

Thus, the Trx- and Grx-mediated RS-NO degradation pathways appear to rely on both transnitrosation (Reaction (11)) and S-thiolation (Reaction (12)) chemistry as part of their catalytic cycles.

**Figure 3:**
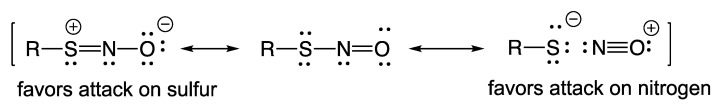
RS-NO resonance forms that favor attack at either sulfur or nitrogen.

Clearly, one of the most important questions to ask at this point is—What governs whether RS-NO reacts with a nucleophilic R’S^−^ at nitrogen (Reaction (11)), transferring the NO^+^ from one thiol to another, or at sulfur (Reaction (12)), forming RSSR’ and HNO? This has been addressed computationally and it is proposed that electronic effects from neighboring groups or proximal protein residues can greatly influence the reaction pathway [54]. That is, the electronic stabilization of the dipoles of one of two ‘antagonistic’ resonance forms by proximal residues can dictate the site of nucleophilic attack (Figure 3).

Clearly, it may also be expected that steric effects play a role as large and hindering R-groups (Figure 3) would likely inhibit nucleophilic attack on sulfur and promote attack on nitrogen. Regardless, it remains possible that the environment (e.g., protein residues) surrounding the RS-NO functional group dictates the reaction pathway. It should also be considered that transnitrosation (Reaction (11)) is an equilibrium process, whereas S-thiolation (Reaction (12)) represents an essentially irreversible process. Thus, even if transnitrosation is a more likely process, given enough time, S-thiolation products may predominate (especially since HNO has numerous other reaction pathways that can essentially remove it from solution) [50].

A reported assay for RS-NO species relies on the selective reduction of RS-NO by ascorbate to the corresponding RSH species [55]. Consistent with this, ascorbate is reported to be capable of degrading RS-NO under physiological conditions [56,57,58], a process greatly accelerated in the presence of copper [37]. The details of the reaction of ascorbate with RS-NO have been examined by several groups. Holmes and Williams [59] reported two distinct pathways for this reaction. One rapid pathway for RS-NO decomposition involves ascorbate and copper while another pathway occurs at high concentrations of ascorbate and in the presence of a copper chelator (i.e., the absence of reactive copper). The copper dependent process is reported to occur via the reduction of RS-NO by cuprous ion giving cupric ion, NO, and the corresponding thiol (Reaction (8)) [37]. The role of ascorbate as well as other reductants in this process is to reduce Cu^2+^ back to Cu^1+^, making copper catalytic. The copper-independent reaction is thought to involve a transnitrosation from RS-NO to ascorbate, giving a nitrosated ascorbate species (an O-nitroso species) that subsequently decomposes to release NO and the ascorbyl radical [59]. Alternatively, an outer-sphere electron transfer mechanism has also been proposed [60]. Regardless, this chemistry is relatively slow (typically <1 M^−1^s^−1^ at physiological pH, although variable depending on the nature of the RS-NO species) [59,60,61] and subject to low yields with other possible products formed. For example, HNO can be a significant product from this reaction [62]. The generation of HNO is proposed to occur via elimination from an O-nitroso ascorbate intermediate species. Regardless, the reaction of ascorbate with RS-NO is potentially ‘messy’ with numerous possible pathways/products and the physiological relevance of these reactions remain unestablished.

## 6. Steady State Levels of RS-NO and Signaling Pathways

Although previous discussion has indicated that RS-NO generation may be slow, inefficient, or biochemically demanding, there appears to be little question that biological RS-NO species exist and their levels can correlate with a variety of pathologies [24,63,64]. Thus, in spite of the issues regarding the chemistry and biochemistry of biosynthesis, it needs to be considered that actual RS-NO levels are a function of the rates of formation AND the rates of degradation. Thus, if RS-NO formation for a particular protein is slow and/or inefficient, its levels can still build up (albeit slowly) if the rates of degradation are even slower or less efficient. That is, the steady state levels of a RS-NO protein can reach high levels or can be built up, in spite of being a slow or improbable process, if available degradation pathways are even slower or less probable. What this may mean is that S-nitrosation is a ‘slow’ signaling process whereby the buildup of S-nitrosated proteins occurs over a protracted period of time and is selective toward proteins or protein thiol sites that are resistant to degradation. This idea, if true, indicates that S-nitrosation as a signaling process is not akin to phosphorylation, which is extremely dynamic, fairly rapid, and specific due to the existence of numerous kinase and phosphatase enzymes [65,66]. Moreover, this idea would predict that the prevalence and/or buildup of RS-NOs may occur very slowly and be involved in processes that do not have a critical and rapid time component (such as is required for neurotransmission or other rapid signaling events). That is, the possible protracted generation of RS-NO formation may be relevant to events that also have a protracted timeline such as the development of certain pathologies (e.g., the progression of neurodegenerative disease, the development of cancers, atherosclerosis, general aging, etc.). Thus, slow/inefficient signaling processes may be relevant to slow or gradual onset biological processes (or slow pathologies). It needs to be emphasized that it is also possible that the “pool” of RS-NO species generated may correlate with a pathology and is not causal to the pathology. It is also possible that as a pathological state increases, the pool of RS-NO also increases and that the RS-NO species represents a protective entity that perhaps acts as a source of NO (or other nitrogen species, *vide infra*) that can be beneficial. Or, it is possible that protein RS-NO formation represents a physiological response to the pathological changes as a means of mitigating the pathology that may occur when inducible nitric oxide synthase (iNOS, NOS2) is induced and significant levels of NO are attainable (e.g., >1 micromolar).

## 7. Hydropersulfides (RSSH) and Possible Importance to RS-NO Degradation

Hydropersulfides (RSSH) and related polysulfur species have recently been proposed as possible important endogenously generated physiological signaling/effector species [67]. It has become increasingly clear that the chemical biology of RSSH is very different from other biologically relevant sulfur species (e.g., RSH, RSSR, sulfenic acid (RSOH), RS(O)OH, etc.) [68,69]. For example, RSS^−^/RSSH is a better nucleophile and reducing agent compared to RS^−^/RSH. As such, it is possible that RSSH can better scavenge potentially toxic and deleterious oxidants and electrophiles compared to RSH and therefore protect cells from these species. Importantly, RSSH is oxidized with respect to RSH (RSSH is at the same oxidation state as RSSR), and therefore could be generated readily under cellular oxidative stress conditions [67]. This presents a unique and possibly important biochemical scenario whereby a superior nucleophile and reductant can be generated under oxidizing (and electrophilic—since many oxidants are electrophilic) cellular stress conditions. This realization led to the hypothesis that RSSH generation could represent a protective physiological response to oxidative and electrophilic stresses [69]. Importantly, numerous studies appear to support this idea [13,70,71,72,73,74].

It is important to understand that RS-NO generation from RSH and NO also represents an oxidation (Reaction (1)) and could be the consequence of an oxidative cellular stress and therefore involved in the etiology of numerous diseases [24,63,75]. If indeed, RS-NO formation contributes to or is involved with disease pathology, then elucidation of the pathways that degrade RS-NO to innocuous species becomes an important topic for the development of potential therapeutic strategies (or physiological protection). It is possible that metabolic conversion of RS-NO back to RSH and NO could represent an ideal process to mitigate disease progression/development.

One recently discovered and currently under-appreciated process that, at first glance, appears to represent a near ideal pathway for RS-NO degradation back to RSH and NO involves RSSH. A relatively recent report indicates that RSSH is capable of reacting with RS-NO, leading to the presumed intermediacy of an S-nitrosodisulfide (RSS-NO), which spontaneously homolyzes under physiological conditions to give NO and the corresponding perthiyl radical (RSS^•^) [76] (Reaction (16)).

R’SSH + RS-NO → [R’SS-NO + RSH] → R’SS^•^ + NO, (16)

2R’SS^•^ → RSSSSR, (17)

**Figure 4:**
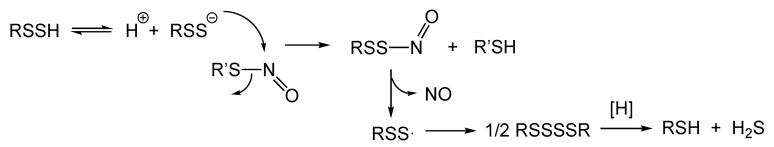
The reaction of RSSH with R’SNO to give NO and, after biological reduction ([H]), RSH and H_2_S.

The RSS· species generated from this reaction is relatively stable, even though it is a paramagnetic radical species, and merely dimerizes to give the dialkyltetrasulfide (RSSSSR) (Reaction (17)) [76,77]. Importantly, unlike the highly oxidizing RS^•^ species, RSS^•^ is relatively non-oxidizing and innocuous with only the propensity, under chemical conditions, to dimerize (and therefore will not initiate any toxicologically troublesome chemistry in a biological system) [76,77]. Moreover, RSS^•^ does not appear to readily react with O_2_, unlike RS^•^ [78], as a solution of RSS^•^ does not consume O_2_ [76]. Thus, RSS^•^ does not interfere with NO or O_2_ signaling/biochemistry. Additionally, it is worth mentioning that polysulfide species have been reported to be subject to enzymatic biological reduction, indicating the possibility of polysulfur species such as RSSSSR being converted back to RSH via well-established enzyme systems such as thioredoxin and glutathione reductase [79,80,81]. Thus, Figure 4 schematically depicts the possible role of RSSH in degrading RS-NO species with the liberation of NO and the eventual regeneration of RSH and concomitant formation of H_2_S.

To be sure, there are sound chemical rationale for the chemistry depicted in Figure 4: (**1**) RSSH is two to four times more acidic than RSH [82,83,84], indicating a higher percentage of the nucleophilic anionic species compared to RSH; (**2**) due to an alpha-effect [85] RSS^−^/RSSH is significantly more nucleophilic than RS^−^/RSH, indicating a facile first reaction [82]; (**3**) the nascent RSS^•^ product (after NO liberation) is very stable and non-oxidizing due to a resonance stabilization of the unpaired electron, although it is capable of dimerization [76,77]; and (**4**) reductive metabolism of polysulfides in general (e.g., RS_n_R, n ≥ 2, R = alkyl, H) to give the corresponding RSH species has been reported [81].

Considering the problematic issues associated with other pathways of RS-NO degradation (*vide supra*, e.g., low reactant concentrations, requirement for light, generation of other reactive species, specificity for GS-NO, etc.), the biochemistry of RSSH-mediated RS-NO degradation appears to be an attractive alternative as an endogenous pathway for lowering general RS-NO levels and, especially, for liberating NO. This may be especially true since RSSH (e.g., GSSH) levels can be significant. For example, in mouse tissues (e.g., heart, liver and brain), GSSH has been reported to be as high as 50–150 micromolars [70]. Moreover, as above-mentioned, the formation of RS-NO from RSH and NO represents an oxidation and is more likely to occur under cellular oxidative stress conditions. Additionally, high levels of NO via the induction of iNOS occurs as a result of inflammatory stress (which can involve an oxidative stress component [86]), which can lead to more facile RS-NO generation (*vide supra*). Importantly, the generation of RSSH from RSH represents an oxidation and is also more likely to occur under cellular oxidative stress [67]. Thus, it seems probable that RS-NO and RSSH will be generated under similar cellular redox environments, possibly indicating that RSSH can protect cells from potentially deleterious RS-NO formation. Considering the previous discussion regarding the likely importance of degradation pathways in determining the steady-state levels of RS-NO, it is possible that an increase in intracellular RS-NO can result from a decreased level of intracellular RSSH.

If RS-NO species are important in the etiology of disease, as has been proposed for neurodegenerative disease [87], cancer [88], and many inflammatory diseases [89,90], it is possible that intracellular RSSH levels are an important factor in determining RS-NO levels and disease status. This may be especially true for protein-RS-NO sites that are not in transnitrosation equilibrium with GSNO or substrates for Trx/Grx, and therefore can potentially build up in concentration.

Although the idea that RSSH species may be a factor in determining the levels of biological RS-NO appears to be viable and worthy of consideration, there remain questions associated with this hypothesis. One obvious question is with regard to the fate of the RSS^•^ species generated after NO release (Reaction (16)). In a purely chemical system, RSS^•^ has been found to simply dimerize to the tetrasulfide (Reaction (17)). However, in a biological milieu, this may not be a primary fate due to the second-order kinetic dependence on RSS· dimerization (i.e., the likelihood of two RSS^•^ species ‘finding’ each other to dimerize should be low at physiological concentrations), especially if other facile first order reactions exist. Thus, if indeed RSSH are involved with the degradation of RS-NO via Reaction (16), what might be the predominate biological fate of RSS^•^? As a paramagnetic one-electron oxidized sulfur species, it is not likely to be a substrate for reducing systems typically involved in the reduction of other diamagnetic oxidized sulfur species such as disulfides, sulfenic acids, RS-NOs, etc. As above-mentioned, since RSS^−^/RSSH are good one-electron reductants, the oxidized species RSS^•^ are not strong oxidants (at least not like the corresponding thiyl radicals, RS^•^) and are more akin to the one-electron oxidized species associated with antioxidants such as ascorbate and the tocopherols [91]. The ascorbyl radical (the one-electron oxidized product of ascorbate and akin to RSS^•^) can be recycled back to fully reduced ascorbate via NADH/NADPH-dependent reductases [92]. Although it is not known whether these ascorbyl radical reducing systems, or others, can recycle RSS^•^ back to RSSH, it is intriguing to speculate that this could be a possibility. It is also possible that other one-electron reductants such as ascorbate or α-tocopherol can reduce RSS^•^ to RSS^−^. The reduction potentials for the ascorbyl radical/ascorbate couple (Asc^−^, H^+^/Asc^−^) and the α-tocopheroxyl/α-tocopherol couple (TO^•^, H^+^/TOH) are 282 mV and 500 mV (pH 7), respectively [32], and a calculated reduction potential for the HSS·/HSS^−^ couple is reported to be 680 mV (pH 7) [93]. These values are consistent with the possibility that either ascorbate or α-tocopherol can reduce RSS^•^ to RSS^−^/RSSH.

## 8. RS-NO as a Storage Form of NO and Regulated NO Release

The degradation of RS-NO may be of importance in the regulation of protein activity and, as alluded to above, potentially relevant to the etiology of numerous diseases. The reduction of RS-NO back to RSH may, for example, restore protein activity and mitigate disease pathology. To be sure, not all RS-NO degradation pathways produce RSH. For example, photochemical degradation produces the reactive RS^•^ and dehydrogenase-mediated degradation can produce other oxidized thiol species (e.g., sulfinamide, sulfinic acid, or disulfide, *vide supra*). Regardless, the nature of the nitrogen oxide product of RS-NO degradation may be equally important. Of particular interest with regard to the RSSH-mediated degradation of RS-NO is the fact that the products are NO and, eventually, RSH. Most other mechanisms of RS-NO degradation do not directly produce NO or are likely physiologically irrelevant. For example, “NO^+^” transfer (transnitrosation) can occur (Reaction (11)), which does not release NO. HNO can also be generated (Reaction (12)), which has biological effects/chemistry distinct from NO, and other reduced nitrogen species such as NH_3_ or NH_2_OH can be produced via dehydrogenase mechanisms. NO can be the product of the Cu^I^-mediated RS-NO degradation (Reaction (8)), but as above-mentioned, this pathway may not be physiologically relevant (or at least not as a general mechanism of NO release). Finally, photochemical production of NO (along with the potentially deleterious RS·, Reaction (9)) from RS-NO degradation is also not generally considered to be physiologically accessible. Thus, in instances where RS-NO may serve as a physiological ‘reservoir’ for NO that can be released at opportune times, RSSH-mediated release appears optimum and unique among all currently considered pathways. To be sure, the idea that RS-NO species are storage forms of NO that can be released in a regulated fashion has been previously and extensively proposed [94,95,96,97,98], albeit without chemically reasonable and regulated proposed mechanisms of NO release. Thus, the idea that RSSH can mediate NO release, if true, may address a major deficiency in the proposals that RS-NO species are storage reservoirs for NO. That is, with no chemically reasonable and facile mechanism for NO release from RS-NO, it is difficult to fully embrace this idea and RSSH species may provide a previously unappreciated and feasible mechanistic pathway.

Clearly, if RSSH species are involved in purposeful and physiologically important NO liberation from RS-NO species, the regulation of RSSH generation becomes an important topic. A detailed discussion of this topic is beyond the scope of this review and many thorough reviews/reports are available [99,100]. However, it is noteworthy that the enzymes involved in RSSH biosynthesis (i.e., cystathionine gamma lyase, CSE, and cystathionine beta synthase, CBS, enzymes more known for their roles in transsulfuration [101]) have other functions and are promiscuous (i.e., can have numerous possible substrates giving various products including Cys-SSH). Importantly, to date, there have not been thorough and systematic studies that examine the factors that regulate the various activities of these enzymes. That is, it seems likely that these versatile enzymes will have a preferred substrate/catalytic reaction depending on, for example, the cellular redox status, phosphorylation state, or other regulatory factors. A recent and provocative report indicates that the biosynthesis of Cys-SSH can also occur via the actions of cysteine tRNA synthetase (CARS) [102]. This study also reports that the Cys-SSH generated from CARS can be translationally incorporated into proteins. As with CSE and CBS, the regulation of this process remains to be determined. Thus, based on the current state of understanding of the regulation of RSSH biosynthesis (as well as RS-NO formation), it is difficult to determine whether RSSH and RS-NO are temporally and/or spatially related. However, it is worth noting that high levels of NO (which will presumably increase the likelihood of RS-NO generation) occur under inflammatory conditions (via the induction of iNOS expression) and Cys-SSH formation from CSE may also be increased under cellular stress conditions [70]. Regardless, examination of the mechanisms of regulation of RSSH biosynthesis remains a pressing issue and will be of paramount importance in ultimately determining the physiological utility and function of the RSSH functional group. It is, however, worth noting that RSSH and H_2_S are intimately linked via an equilibrium reaction (Reaction (18)), which indicates that the presence of H_2_S can be used as a marker for RSSH (and vice-versa) [103,104]. Thus, studies examining H_2_S bioactivity may also involve RSSH species.

H_2_S + RSSR ⇌ RSSH + RSH, (18)

## 9. H_2_S/RS-NO Interactions and ONSS^•^ Formation

Thus far, there has been an emphasis on a discussion of the chemical biology of RSS-NO, a key intermediate in the RSSH-mediated liberation of NO from RS-NO (Figure 4). It may be expected that RSS-NO and HSS-NO (simply RSS-NO with R = H) can have similar properties and that HSS-NO can be generated from an analogous process whereby HSS^−^ reacts with RS-NO (Reaction (16), where R’ = H). Significantly, it has been reported that the generation of ONSS^−^/ONSSH can occur as a result of the reaction of RS-NO with H_2_S. Clearly, ONSS^−^ formation cannot occur via a simple bimolecular process and must occur via a series of reactions. It is proposed that an initial reaction of HS^−^/H_2_S with RS-NO generates thionitrous acid (HSNO), which is a key intermediate in the formation of ONSS^−^ (Reaction (19)). Subsequent reaction of HSNO with H_2_S then produces H_2_S_2_ (and HNO), which can further react with either RS-NO or HSNO to give ONSS^−^ (Reactions (20)–(22)) [105,106].

H_2_S + RS-NO → RSH + HSNO, (19)

H_2_S + HSNO → H_2_S_2_ + HNO, (20)

H_2_S_2_ + RS-NO → ONSS^−^ + RSH + H^+^, (21)

H_2_S_2_ + HSNO → H_2_S + ONSS^−^ + H^+^, (22)

The inorganic polysulfide H_2_S_2_ intermediate in the above chemistry is analogous to RSSH and therefore the chemistry proposed above appears reasonable. To be sure, previous work has shown that inorganic hydropersulfides and hydropolysulfides (i.e., HSS_n_^−^, n ≥ 1) are prevalent and likely relevant biological sulfur species [102]. It is expected that HSS^−^ can perform analogous chemistry to that shown above for the RSS^−^ species. That is, HSS^−^ should be a potent nucleophile (superior to HS^−^) similar to RSS^−^, and HSS^•^ (or ^−^SS·/HSS^•^) should be relatively stable compared to ^−^S^•^/HS^•^. The chemical rationale for these statements is identical to those previously discussed for the analogous RSS^−^/RSSH species.

As discussed previously, the S–N bond in RSS-NO species is relatively weak and homolytic cleavage of the S–N bond (Reaction (16)) has been shown to occur readily due to the relative stability of the two paramagnetic products NO and RSS^•^. Not unexpectedly, facile homolytic cleavage of the S–N bond of ONSS^−^ has also been proposed [105] and as such, ONSS^−^ is hypothesized to be an effective NO-donor molecule [107]. However, this idea has been disputed. ONSS^−^ has been reported to be very unstable and generates, instead of NO, HNO and inorganic sulfur species [108]. Others have also reported that ONSS^−^ is very stable and not a good NO donor except in the presence of hemeproteins with open coordination sites (e.g., methemoglobin, deoxyhemoglobin) [109]. Computational studies indicate that the S–N bond in the anionic ON-SS^−^ species is shorter, and therefore stronger, than the S–N bond of RSS-NO, indicating that the anionic ONSS^−^ has increased stability with regard to homolytic cleavage to NO compared to RSS-NO [76]. Further examination of ONSS^−^ biochemistry indicates that it is resistant to reduction by biological NADPH-dependent reducing systems (e.g., Trx reductase, GSH reductase) [106] as well as resistant to reduction by thiols [107] and therefore can serve as a relatively long-lived source of biologically active polysulfides and nitrogen oxides. It is important to realize that the chemical reactivity of ONSS^−^ will be closely analogous to RSS-NO only when in the neutral, protonated form, ONSSH, and therefore the pK_a_ of ONSSH (estimated to be approximately 5 [110]) and the pH of the system become important factors in its chemical fate.

The biological relevance of endogenous ONSS^−^ formation remains to be established. Although there is little question that RS-NO and H_2_S are endogenously formed and present in biological systems, one formidable issue with regard to the formation of ONSS^−^ is that it requires numerous steps that involve fleeting species (i.e., Reactions (19)–(22)) and this scenario may preclude significant physiological ONSS^−^ generation from this chemistry. Regardless, further investigation of the chemical and biological properties of ONSS^−^/ONSSH is warranted.

## 10. Summary

The primary target for NO signaling is the enzyme sGC, which catalyzes the conversion of GTP to cGMP. This NO/sGC/cGMP signaling system is an important aspect of the regulation of the cardiovascular system (as well as other physiologies associated with smooth muscle activity). Another proposed mechanism of NO-mediated cellular signaling is via the generation of NO-derived RS-NOs in proteins. There is little question that RS-NOs are present and ubiquitous in biological systems. However, the mechanisms by which they are made and/or degraded remain unestablished and therefore the regulation of RS-NO formation as a signaling paradigm remains obscure. Regardless, it appears that there are currently two primary aspects of RS-NO formation/signaling:

(1) RS-NO are species whose levels can build up slowly and correlate with numerous disease states (e.g., neurodegenerative diseases, cancer). Although in most cases it is not completely understood how RS-NO levels relate to the etiology of disease, it is speculated in some cases that RS-NO levels can be key factors in disease progression via alteration of thiol protein function and/or properties. Additionally, RS-NO formation in proteins may represent normal physiological signaling phenomenon, akin to other protein regulation processes that involve thiol redox species.

(2) RS-NO species can be storage forms for NO that are purposefully released when NO is needed. This may be especially true in the cardiovascular system where NO is an established regulator of many physiological homeostatic mechanisms.

**Figure 5:**
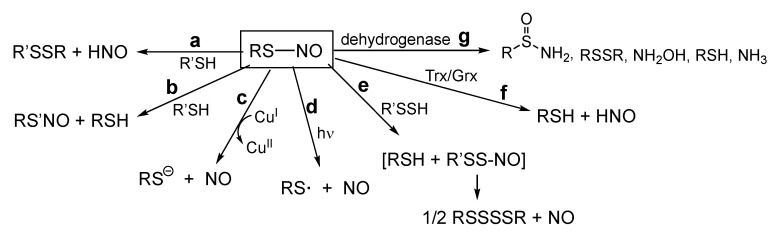
Currently proposed mechanisms for RS-NO degradation. (**a**) S-thiolation and the generation of HNO, (**b**) transnitrosation, transfer of ‘NO^+^’ with no generation of NO, (**c**) Cu^I^-mediated reaction with formation of NO and RSH (not likely to be of general relevance based on current literature), (**d**) photochemical cleavage of the RS-NO bond with formation of NO and highly oxidizing RS·(not of general relevance except maybe in specific situations (e.g., skin)), (**e**) RSSH-mediated destruction of RS-NO with formation of NO and unreactive RSS· (which dimerizes to RSSSSR, which can be reduced to RSH), (**f**) Trx or Grx-mediated reduction of RS-NO to RSH and ultimately to HNO, not NO, (**g**) dehydrogenase-mediated conversion of RS-NO to a variety of possible products without generation of NO, primarily relevant only to GS-NO.

Although there are currently numerous possible chemical/biochemical mechanisms for the formation and degradation of RS-NO species, many of these are fraught with issues that may preclude their biological relevance or accessibility (*vide supra*). As discussed herein, a newly discovered chemical reaction between RSSH and RS-NO is capable of liberating NO and eventually forming RSH from RS-NO and considering the fact that numerous reports indicate the presence and prevalence of RSSH species in biological systems, this reaction may represent an ideal and endogenous mechanism for regulating RS-NO biology/signaling. Especially intriguing is the fact that the RSSH/RS-NO reaction generates NO (as well as regeneration of a reduced thiol) and, therefore, may be ideal for the rapid liberation of NO from RS-NO (point #2 above). Importantly, other mechanisms of RS-NO degradation that may be potentially relevant to biological systems do not liberate NO. For a brief overview, Figure 5 depicts the currently proposed mechanisms for RS-NO degradation and briefly describes their products. The possible biological relevance and utility of many of these processes are discussed earlier.
